# Evolutionary implications of C_2_ photosynthesis: how complex biochemical trade-offs may limit C_4_ evolution

**DOI:** 10.1093/jxb/erac465

**Published:** 2022-11-28

**Authors:** Catherine A Walsh, Andrea Bräutigam, Michael R Roberts, Marjorie R Lundgren

**Affiliations:** Lancaster Environment Centre, Lancaster University, Lancaster LA1 4YQ, UK; Faculty of Biology, Bielefeld University, Universität str. 27, D-33615 Bielefeld, Germany; Lancaster Environment Centre, Lancaster University, Lancaster LA1 4YQ, UK; Lancaster Environment Centre, Lancaster University, Lancaster LA1 4YQ, UK; University of Illinois, USA

**Keywords:** C_2_ photosynthesis, C_3_–C_4_ intermediates, C_4_ evolution, carbon-concentrating mechanism, C:N balance, GABA, glycine shuttle, nitrogen sink, photorespiration, serine, tricarboxylic acid pathway

## Abstract

The C_2_ carbon-concentrating mechanism increases net CO_2_ assimilation by shuttling photorespiratory CO_2_ in the form of glycine from mesophyll to bundle sheath cells, where CO_2_ concentrates and can be re-assimilated. This glycine shuttle also releases NH_3_ and serine into the bundle sheath, and modelling studies suggest that this influx of NH_3_ may cause a nitrogen imbalance between the two cell types that selects for the C_4_ carbon-concentrating mechanism. Here we provide an alternative hypothesis outlining mechanisms by which bundle sheath NH_3_ and serine play vital roles to not only influence the status of C_2_ plants along the C_3_ to C_4_ evolutionary trajectory, but to also convey stress tolerance to these unique plants. Our hypothesis explains how an optimized bundle sheath nitrogen hub interacts with sulfur and carbon metabolism to mitigate the effects of high photorespiratory conditions. While C_2_ photosynthesis is typically cited for its intermediary role in C_4_ photosynthesis evolution, our alternative hypothesis provides a mechanism to explain why some C_2_ lineages have not made this transition. We propose that stress resilience, coupled with open flux tricarboxylic acid and photorespiration pathways, conveys an advantage to C_2_ plants in fluctuating environments.

## Teaser

An alternative theory for bundle sheath ammonia tolerance in C_2_species suggests that C_2_to C_4_evolutionary transitions may depend on bundle sheath nitrogen hub optimization.

## Introduction

**Fig. 1.**
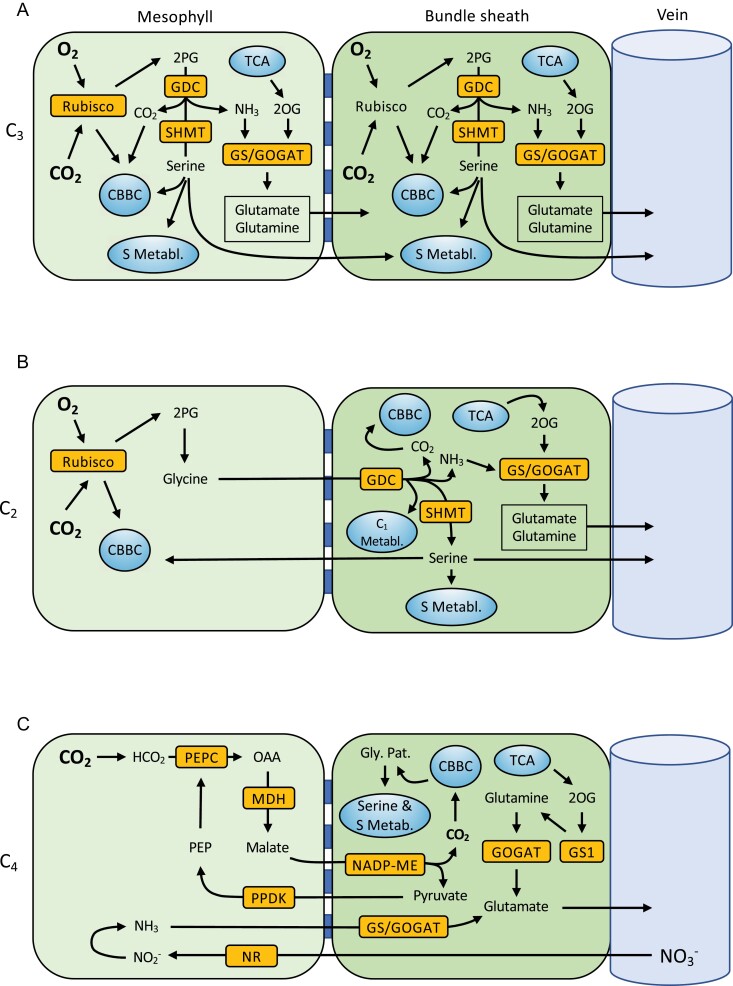
Carbon and nitrogen pathways in C_3_, C_2_, and C_4_ species. (A; C_3_) and (B; C_2_) show photorespiration as the principal source of serine in C_3_ and C_2_ species, respectively. Mesophyll cells will carry out the bulk of central metabolism in C_3_ species (A), with minimal input from bundle sheath cells as a result of their fewer organelles (C_1_ metabolism not shown). (C) shows an absence of photorespiration and depicts the non-phosphorylated glycerate pathway (Gly. Pat.) in the cytosol as a viable serine source for C_4_ species, although the phosphorylated pathway in the chloroplast may also play a role (not shown) (C is adapted from Igamberdiev and Kleczkowski, 2018; Jobe *et al.*, 2019). Integration with sulfur metabolism (S Metab.) is also shown. Orange blocks represent fundamental enzymes. Blue blocks in between mesophyll and bundle sheath cells represent plasmodesmata. Calvin–Benson–Bassham cycle (CBBC), tricarboxcylic acid cycle (or pathway) (TCA); 2-phosphoglycolate (2-PG); 2-oxoglutarate (2-OG); oxaloacetate (OAA); phosphoenolpyruvate (PEP). Enzymes: glycine decarboxylase complex (GDC), serine hydroxymethyltransferase (SHMT), glutamine synthetase/glutamine:2-oxoglutarate aminotransferase (GS/GOGAT), phospho*enol*pyruvate carboxylase (PEPC), malate dehydrogenase (MDH), NADP-malic enzyme (NADP-ME), pyruvate-orthophosphate dikinase (PPDK), glutamine synthetase 1 (GS1), amd nitrate reductase (NR).

C_2_ photosynthesis is a carbon-concentrating mechanism (CCM) that recovers and reassimilates CO_2_ released from photorespiration to attenuate C losses, such as those seen in C_3_ species under similar environmental conditions (Schlüter and Weber, 2016). Unlike the C_4_ photosynthesis CCM, that dramatically reduces rates of photorespiration, the C_2_ photosynthetic pathway relies on it and, in doing so, achieves a potentially ideal relationship with photorespiration. It benefits from the possible positive aspects of photorespiration (e.g. Timm and Bauwe, 2013; Busch *et al.*, 2018; Eisenhut *et al.*, 2019), while minimizing its negative impacts on net C assimilation efficiency under warm and dry conditions (Sage *et al.*, 2013). The success of C_2_ photosynthesis hinges upon the confinement of photorespiratory glycine decarboxylation to an inner leaf compartment, the bundle sheath (BS) (Fig. 1). C_2_ plants assimilate CO_2_ via the Calvin–Benson–Bassham (CBB) cycle and initiate the photorespiratory cycle in the mesophyll. However, because C_2_ plants uniquely confine functional glycine decarboxylase complex (GDC) enzymes to the BS, photorespiratory glycine that is produced in the mesophyll must diffuse into the BS to be decarboxylated, which releases and concentrates CO_2_ for effective reassimilation into a BS CBB cycle, liberates ammonia (NH_3_) and synthesizes serine in this inner compartment (Fig. 1 and discussed in detail below; Rawsthorne *et al.*, 1988). We propose that this phenotype conveys four key benefits in C_2_ compared with C_3_ plants, namely an expanded ecological niche into warmer and drier climates, enhanced net C assimilation due to better C reclamation and concentration, improved stress tolerance, and reduced nutrient losses that result from the C dilution effect experienced under elevating atmospheric CO_2_ concentrations. Despite these potential benefits, relatively little is known about this rare C_2_ pathway compared with other types of photosynthetic metabolism. To shed light on these potential benefits conveyed by C_2_ physiology, we discuss what is currently known and, where unknown, propose hypotheses that warrant further investigation.

## Diversity and distribution of C_2_ photosynthesis

C_2_ photosynthesis has been characterized in >50 species of herbaceous and semi-woody shrubs (but not trees), originating from 20 plant lineages (4 monocot and 16 eudicot) representing 11 plant families (Sage *et al.*, 2018; Lundgren, 2020). Fifteen of these 20 C_2_ lineages also contain species using C_4_ photosynthesis, while five C_2_ lineages lack close C_4_ relatives.

With far less than 1% of plant species currently identified as using C_2_ photosynthesis, it is comparatively much rarer than C_3_ and C_4_ types that are used by >95% and ~3% of global plant species, respectively. Despite so few species using this rare physiology, C_2_ plants are remarkably geographically and ecologically widespread, inhabiting diverse environments across every continent except Antarctica (Lundgren and Christin, 2017). The biogeography of C_2_ species reveals an overall preference for regions with high light, heat, and possible drought (i.e. environments that typically induce high rates of photorespiration). However, C_2_ species will also successfully establish in particularly wet, cold, and shady environments (e.g. Powel, 1978; Christin *et al.*, 2011; Lundgren *et al.*, 2015; Khoshravesh *et al.*, 2016; Lundgren and Christin, 2017). Interestingly, C_2_ species from lineages that lack C_4_ relatives are among the most geographically widespread and are more likely to inhabit cooler, wetter environments with richer soils than C_2_ lineages with C_4_ relatives (Lundgren and Christin, 2017). Overall, the broad ecological distribution of C_2_ plants points to a suite of adaptations particular to the C_2_ phenotype, which might differ between lineages that did or did not also evolve C_4_ photosynthesis.

Current hypotheses suggest that the complex trade-offs between C and N in the C_2_ phenotype convey strong selection pressure for a C_4_ photosynthetic pathway to evolve (Monson and Rawsthorne, 2000; Mallmann *et al.*, 2014; Bräutigam and Gowik, 2016; Schlüter and Weber, 2016). Indeed, the C_2_ phenotype is often associated with its role in facilitating the evolution of the complex C_4_ photosynthetic state, having been repeatedly identified in C_3_–C_4_ intermediate species (i.e. evolutionary intermediate species with phenotypes that fall between that of typical C_3_ and C_4_ plants) (Schlüter *et al.*, 2017; Sage, 2021). This is not the case for all C_2_ lineages, however, as C_2_ photosynthesis has been identified as existing for very long periods of time (e.g. >10 million years; Christin *et al.*, 2011) in those lineages where C_4_ photosynthesis never emerged (Lundgren and Christin, 2017), suggesting that it can be a stable evolutionary state and not always an inevitable path to C_4_ photosynthesis (Edwards, 2019; Lundgren, 2020, Lyu *et al.*, 2022). This notion of a stable-state C_2_ phenotype is supported by constraint-based modelling, which identifies the C_2_ phenotype as an optimal metabolic solution under a defined set of resource limitations (Blätke and Bräutigam, 2019). However, it remains unclear why some C_2_ lineages transition to adopt C_4_ physiology while others do not, and to what extent their broad ecological tolerance plays a role.

C_2_ plants have an inherent physiological plasticity that may allow them to inhabit such large ecological ranges. Because the glycine shuttle only activates under environments that promote photorespiration, C_2_ plants will be physiologically similar to C_3_ plants under non-photorespiratory conditions (e.g. cool, wet environments): that is, they will both engage the CBB cycle in the mesophyll cells. However, under warm, arid, and high light conditions, CO_2_ concentrations within the leaf wane, which increases the oxygenation to carboxylation ratio of Rubisco, and consequently rates of photorespiration and the glycine shuttle in C_2_ plants (Schulze *et al.*, 2016). Therefore, any negative effects of photorespiration on plant growth and yield that result from C losses should be less pronounced in C_2_ compared with C_3_ plants. Thus, in theory, C_2_ species could thrive largely on a mesophyll CBB across a range of environments, supported by a C_2_ pathway only when challenging conditions arise. Despite these theoretical advantages of physiological plasticity and optimized utilization of photorespiration, the relative rarity of C_2_ species suggests that there might be as yet unidentified significant costs to this phenotype or a gross underestimation of the ubiquity of C_2_ physiology. Indeed, if C_2_ plants do encounter higher photorespiratory flux compared with C_3_ plants—which remains unconfirmed in the literature—then they may be more common in hot, dry environments than previously thought. More work is needed to identify whether the C_2_ phenotype exists across a broader range of species than is currently known.

## Carbon assimilation

In both C_3_ and C_2_ plants, C is assimilated via the CBB cycle in mesophyll cells when Rubisco catalyses the addition of CO_2_ to ribulose 1,5-bisphosphate (RuBP) (Fig. 1A, B). Rubisco will also catalyse a reaction of O_2_ with RuBP, which creates toxic by-products that need to be detoxified through photorespiration (Fig. 1A, B). In C_3_ plants, the entire photorespiratory pathway occurs within a single cell type, usually the mesophyll. The C_2_ CCM, however, functions by spreading photorespiration across mesophyll and BS cells. Specifically, the GDC enzyme is exclusively localized to the BS of C_2_ plants, such that any glycine that is produced during photorespiration builds up in the mesophyll, creating a concentration gradient from which glycine diffuses into the BS (Monson and Rawsthorne, 2000). Once in the BS, GDC activity—coupled with serine hydroxymethyltransferase (SHMT) using tetrahydrofolate/5,10-methylene tetrahydrofolate as a cofactor—catalyses the release of CO_2_, along with NH_3_ and serine (Fig. 1B) (Sage *et al.*, 2012). This effective CCM approximately triples intraplastidial CO_2_ concentrations compared to closely related C_3_ species (Keerberg *et al.*, 2014). Indeed, by running a mesophyll C_3_ photosynthetic cycle in parallel with the dual-cell glycine shuttle, C_2_ plants can recycle CO_2_ lost from photorespiration more effectively than C_3_ plants (Sage *et al.*, 2012). Some C_3_ species, such as rice, have developed effective single-celled strategies to reclaim photorespiratory CO_2_ release in climates where rates of photorespiration are high (Sage and Sage, 2009). However, despite minimizing CO_2_ losses, these C_3_ species fail to achieve both the elevated photosynthetic rate and low CO_2_ compensation points that C_2_ plants do, which tend to be intermediate between those measured in C_3_ and C_4_ species (Vogan *et al.*, 2007).

**Fig. 2.**
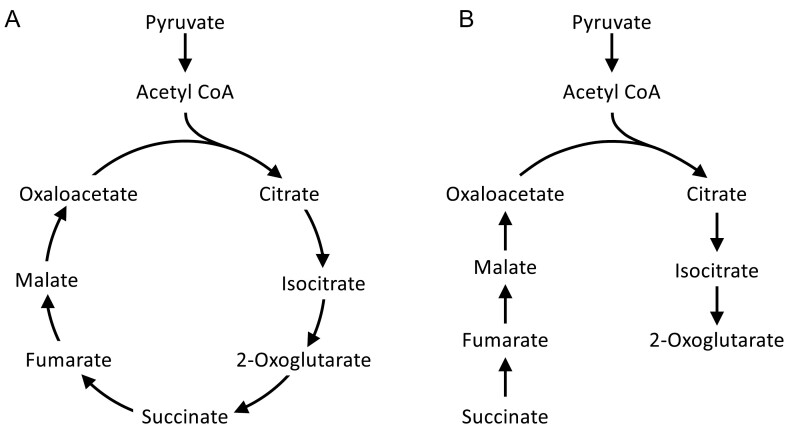
The tricarboxylic acid (TCA) flux modes. The cyclic mode (A) and the non-cyclic dual branched pathway (B). Our C_2_ mechanism hypothesis suggests that the traditional cyclic mode may operate in the dark similarly to C_3_ species (some light operation will still occur), while a non-cyclic pathway may be operational in the light under photorespiratory conditions within C_2_ species (malate and citrate valves not shown).

Successful establishment of the C_2_ CCM relies on adaptations to leaf anatomy that underpin the biochemical changes essential for metabolic remodelling. This is evident in the higher leaf vein densities that have evolved in some lineages as a prerequisite for C_2_ pump establishment, which not only enables efficient metabolite exchange between mesophyll and BS cells, but also maintains hydraulic flow under elevated evapotranspiration (Sage *et al.*, 2011). Efficient decarboxylation of glycine in the C_2_ BS also requires both an increase and realignment of BS chloroplast, mitochondria, and peroxisome organelles compared with those in mesophyll cells to accommodate the enhanced influx (Khoshravesh *et al.*, 2016). The proliferation of BS mitochondria specifically not only facilitates CO_2_ release from glycine decarboxylation, but may also improve biosynthetic capacity, for example through a non-cyclic, dual branched tricarboxylic acid (TCA) pathway, as discussed in detail below (Fig. 2B) (Tcherkez *et al.*, 2009; Sweetlove *et al.*, 2010). It is worth noting that because C_3_ plants have functional GDC in both mesophyll and BS cells, they could, in theory, also translocate glycine between the two cell types. This seems unlikely, however, as it would be more parsimonious for the glycine to pass through the abundant GDC/SHMT in the mesophyll, rather than unnecessarily translocating to the BS to be decarboxylated. Furthermore, mesophyll glycine levels would likely not concentrate high enough to permit diffusion into the BS. There are also many fewer organelles within C_3_ BS cells, which would limit decarboxylation capacity within this cell type should any glyine reach the BS.

While the literature often highlights the negative impacts of photorespiration (e.g. Walker *et al.*, 2016; Fernie and Bauwe, 2020), it is also an essential and beneficial metabolic pathway (Timm and Bauwe, 2013; Busch *et al.*, 2018; Eisenhut *et al.*, 2019). It has even been suggested that photorespiration may not convey CO_2_ losses but instead show regulatory atmospheric advantages (Tolbert, 1994). Of course, any effects produced by photorespiration will be flux dependent. For example, at a 25–30% O_2_ fixation rate, flux through the C_2_ cycle can be as high as one-third the flux through Rubisco (Sharkey, 1988). As a consequence, the flux into NH_3_ and through C_1_ metabolism and serine can also be up to one-third the flux through Rubisco, making these among the highest metabolic fluxes in the plant, depending on species type and environmental conditions (Busch, 2020). Therefore, plants using C_3_ or C_2_ photosynthesis, but not C_4_ photosynthesis, will have appreciable flux through photorespiration and thus into NH_3_ and serine and through C_1_ metabolism (Mouillon *et al.*, 1999; Bauwe *et al.*, 2010; Schulze *et al.*, 2016; Jardine *et al.*, 2017).

In summary, the C_2_ glycine shuttle transports CO_2_, NH_3_, and serine into the BS, and necessitates high flux through C_1_ metabolism (Fig. 1B). In any angiosperm plant, the BS is the gateway between the leaf and the remainder of the plant. Through the BS, leaves export sucrose to move assimilated C, the amino acids glutamate, glutamine, aspartate, asparagine, and notably serine to distribute assimilated N (Fig. 1B). The C:N ratio in the foliar compartment can be broadly approximated to 10:1 (Hager *et al.*, 2016). If photorespiration runs at 30% of C fixation, each C fixed results in 0.15 ammonia produced which are detoxified using glutamate and glutamine. Since serine and glycine are directly produced in photorespiration and glutamate and glutamine are produced during ammonia refixation, all N that is exported from the leaf to support the remainder of the plant may be supplied via the C_2_ glycine shuttle (e.g. as per data reported in Wilkinson and Douglas, 2003). Therefore, one needs to postulate that the stream of metabolites out of GDC/SHMT is split into those returning to the mesophyll, entering the veins to be distributed throughout the plant, and serving as substrates in the BS itself. To understand the evolutionary implications of the C_2_ pathway, one consequently needs to consider the possible advantages afforded to C_2_ plants beyond mere increased efficiency in C assimilation.

## Nitrogen assimilation

It is often assumed that the NH_3_ released into the BS from the C_2_ glycine shuttle would return to the mesophyll to complete the photorespiratory cycle (Rawsthorne *et al.*, 1988; Mallmann *et al.*, 2014; Bräutigam and Gowik, 2016), similar to the way that amino acids (e.g. alanine) or organic acids (e.g. pyruvate) are used to balance N between the mesophyll and BS cells of C_4_ plants. However, a complex coordination of photosynthesis, respiration, and photorespiratory N assimilation, similar to that occurring within the mesophyll cells of C_3_ plants (Stitt and Krapp, 1999), could also be present in the BS of stable-state C_2_ species, foregoing parts of the N shuttle previously described in C_2_ species from lineages with C_4_ relatives. (Rawsthorne *et al.*, 1988; Monson and Rawsthorne, 2000). Leaf C and N use efficiencies in C_2_ leaves lie largely in between those of C_3_ and C_4_ species (Vogan and Sage, 2011), which suggests that the C to N balance must provide an advantage to C_2_ individuals over C_3_ plants under certain environmental conditions, and that NH_3_ cycling has concurrently been managed in accordance with C gain. Notably, the further down the evolutionary trajectory towards C_4_ metabolism a particular C_2_ phenotype is, the greater its C and N use efficiencies are realized (Vogan and Sage, 2011; Sage *et al.*, 2018). Indeed, Sage (2021) proposes that the main evolutionary driver for C_4_ photosynthesis may be NH_3_ recovery and reassimilation, rather than C acquisition, as has been the dominant thinking in the field for decades. This scenario could present itself when C_3_ plants are subjected to frequent periods of high photorespiratory flux, which require adjustment to their N stoichiometry. Consequently, this restructuring of N metabolism could allow the co-evolution of the stable state C_2_ species alongside C_4_ phenotypes (Lyu *et al.*, 2022), resulting in a ‘super C2’ phenotype in these few lineages that could optimise both their resource use and environmental range.

When CO_2_ concentrations increase in the BS, C_2_ plants must also adjust their N stoichiometry; one theory on how this could be achieved is by using either side of a dual branched TCA pathway (Fig. 2B). This accommodates for increases to either C or N, by making amino acids if N is higher or organic acids if C is too high. These efforts could improve the N use efficiency (NUE) of C_2_ plants through acclimatory transitions that redistribute N allocation throughout the plant, as is found in C_3_ plants (Stitt and Krapp, 1999).

**Fig. 3.**
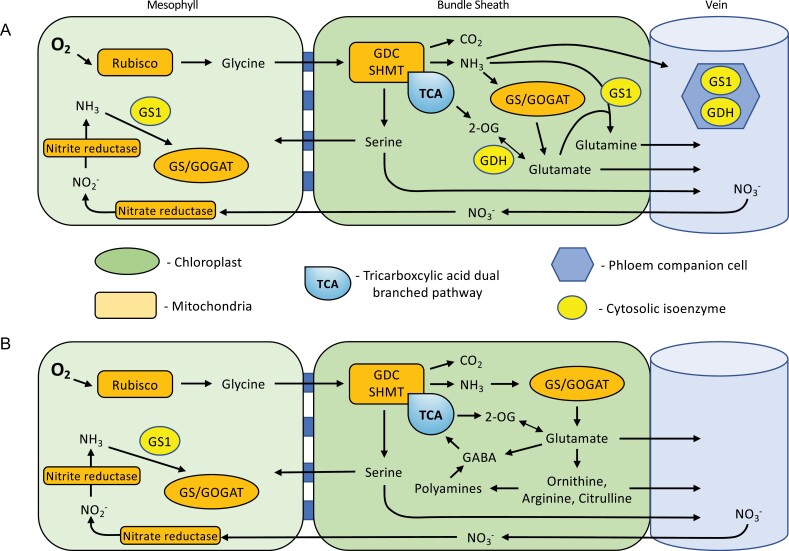
Hypothetical glutamate feed depicting the leaf N hub in stable state C_2_ species. (A) Glutamine synthetase (GS1) and glutamate dehydrogenase (GDH) isoenzymes are located in both the cytosol and companion cells. These mechanisms could work to detoxify NH_3_ produced following glycine decarboxylation, with possible signals from H_2_O_2_ and a potential increase of vein density compared with C_3_ relatives facilitating rapid removal of NH_3_ and distribution of nitrogen into non-toxic amino acids. This scenario may work independently or co-dependently with amino acid synthesis (as shown in B), in addition to 2-oxoglutarate (2-OG) and γ-aminobutyric acid (GABA) feeds into the TCA pathway (glutamate–2-OG interconversion may operate in cyclic cooperation with GS/GOGAT according to Stitt *et al.* (2002). Rapid nitrogen transport through the vein to sink tissue may be via glutamate, ornithine, arginine, and citrulline, by cytosolic relocation of metabolites and enzymes.

The need for photorespiratory glycine to be returned to the C_2_ mesophyll should not be ruled out, nor should nitrate sequestration within the vacuole. Yet, evidence from the C_2_ salad crop wild rocket/arugula (*Diplotaxis tenuifolia*), which must undergo rigorous leaf nitrate assessment before sale, suggests that it may be the C_2_ mechanism causing the crop to hyperaccumulate nitrate within the leaf, which frequently exceeds safe levels for human consumption (Weightman *et al.*, 2012; Santamaria *et al.*, 2002). This characteristic would suggest a prominent role for nitrate within C_2_ physiology, that cannot be said for C_3_ salad crops, whose leaf nitrate levels lie well beneath those of wild rocket in the same environment (Signore *et al.*, 2020). Given that wild rocket lacks C_4_ relatives, this species is a likely candidate for the ‘super C2’ metabolism proposed above. If nitrate reduction enzymes are restricted to the mesophyll of C_2_ plants, as they are in C_4_ plants (Jobe *et al.*, 2019), this would allow for a novel N feed into glycine (Fig. 3) and justify the abnormally high nitrate levels common to wild rocket (Iammarino *et al.*, 2022). However, this would be in complete contrast to the high NUE efficiency that is associated with many C_4_ species. Alternatively, as previously mentioned, tight coordination between other metabolic pathways with photorespiration functioning in an open mode rather than a closed cycle could maintain glycine pools necessary for cellular processes within the mesophyll cell. It is also possible that NH_3_ (or NH_4_^+^, depending on pH levels) may simply diffuse from the C_2_ BS directly into the mesophyll, removing the need for complicated stoichiometry.

Similarly to C_3_ species, the NH_3_ generated by the glycine decarboxylation reaction in C_2_ plants must be rapidly assimilated to prevent accumulation and consequent cell damage. Cellular impairment can result from a rise in NH_3_ levels, which causes pH levels to become unmanageable (Bittsánszky *et al.*, 2015). More importantly, NH_3_ is also a known uncoupler of thylakoid electron transport, which directly restricts photosynthetic capacity (Stitt *et al.*, 2002). With shifts in pH, the NH_3_ released from GDC will convert to NH_4_^+^, the accumulation of which is also highly toxic and requires amelioration (Bittsánszky *et al.*, 2015). Because photorespiratory flux should be similar in C_2_ and C_3_ species, running at 30% of Rubisco reactions (Sharkey, 1988), C_2_ phenotypes must also have efficient NH_3_ and NH_4_^+^ remediation mechanisms to mitigate toxicity. The plastidal GS/GOGAT (glutamine synthetase and glutamine:2-oxoglutarate aminotransferase) system, with potential contributions from C_4_-related metabolites, is one possible route for rapid re-assimilation (Rawsthorne *et al.*, 1988; Mallmann *et al.*, 2014; Bräutigam and Gowik, 2016) (Fig. 1C). The spatial segregation inherent to the C_2_ phenotype may provide additional buffering. By isolating GDC in the BS, NH_3_ production is separated from photosynthesis occurring within the mesophyll cells. This separation may provide a benefit for both cell types, whereby the mesophyll must no longer contend with photorespired NH_3_, while the BS Rubisco gets CO_2_ enrichment with an unknown existing mechanism ameliorating NH_3_ prior to accumulation. In theory, the C_2_ CCM must simultaneously act as an N concentration mechanism, that may rely on a soil nitrate feed into the mesophyll under photorespiratory conditions (Fig. 6) or alternatively relinquish previously stored nitrate from the vacuole.

An alternative pathway to alleviate pressure on plastidal GS/GOGAT assimilation is to employ a secondary cycle in the cytosol to fix NH_3_ to supply glutamine synthesis, using an isoenzyme of glutamine synthetase (GS1) (Fig. 3A). It has been found that the cytosolic GS1 enzyme is up-regulated in response to sudden increases in NH_3_ influx to prevent toxicity (Guan *et al.*, 2016). Pérez-Delgado *et al.* (2015) also identified an increase in gene expression of cytosolic GS1 in response to photorespiratory NH_3_, which was found to be concomitant with glutamate dehydrogenase (GDH) expression for glutamate–2-oxoglutarate (2-OG) interchange in the absence of the plastidal GS2, similar to findings by Ferreira *et al.* (2019). Additionally, asparagine synthetase gene expression was also elevated under photorespiratory conditions, suggesting that asparagine serves as another player in N mobilization under stress (Pérez-Delgado *et al.*, 2015). It is likely, however, that there are several GS isoenzymes that function to maintain cellular GS activity, which are regulated in response to levels of cellular N and nutritional type, especially within the vasculature (Bernard and Habash, 2009).

Under photorespiratory conditions, the influx of NH_3_ could be fundamental for C_2_ plants, as the NH_3_ molecule influences the regulation and distribution of multiple GS1 isoenzymes in response to plant N status and environmental stimuli in some C_3_ species, thus providing a protective role through N remobilization (Bernard and Habash, 2009). Indeed, Hirel *et al.* (1983) found significant GS1 activity in both C_2_ and C_4_ species of *Panicum* that was notably absent in the C_3_ phenotype and directly correlated GS1 content with photosynthetic type. Frequent periods of high photorespiratory flux could induce GS1 expression in mesophyll and BS cell types of stable-state C_2_ species by mitigating NH_3_ accumulation via the sharing of N assimilation between both cytosolic and companion cell GS1 and the chloroplastic GS2. Collectively increasing BS N metabolism activity in close proximity to the vasculature (and potentially also coupled with an increase in vein density) means that the C_2_ BS will supply a substantial amount of amino acids to the phloem stream (Weibull *et al.*, 1990; Sandstrom and Pettersson, 1994; Leegood, 2008). This export gateway of the leaf acts as a potential compensatory C_2_ feature to support plastidal GS2 activity under photorespiration. The resulting excess glutamine (from GDH amination) can then act as an export substrate as well as the main substrate for ornithine, arginine, and polyamines (Majumdar *et al.*, 2016) once homeostasis of the cellular glutamine pool is achieved (Fig. 3B). A good proportion of the glutamine could then be exported to improve N mobilization for developmental processes. This export to the phloem opens any auxiliary cycle that may have operated (e.g. any smaller metabolic pathway in which it acts as an amino group donor for growth) and, consequently, necessitates *de novo* 2-OG synthesis. Any *de novo* synthesis of 2-OG from citrate in turn releases additional CO_2_ into the BS. A proportion of the aspartate produced should also be exported to the phloem and removed from any future cycles, necessitating *de novo* C backbone synthesis. These export pathways to the phloem underscore the critical role of the mitochondria to supply C backbones for amino acid synthesis in both C_2_ and C_3_ plants.

In all plants, glutamate and glutamine are also precursors to proline, ornithine, and arginine synthesis, and therefore also to polyamine biosynthesis (Fig. 3B) (Winter *et al.*, 2015). Ornithine is a light-stable amino acid that often interchanges with citrulline. The role of citrulline has not been previously linked to C_2_ species but could be a strategic molecule for the phenotype, as citrulline and ornithine have been shown to accumulate under photorespiration and low concentrations of CO_2_ (Blume *et al.*, 2019). Research in C_3_ species has shown that citrulline is a major long-distance N transporter involved in nutrient release to maintain osmotic pressure gradients, moving from source to sink tissue under stress (Kawasaki *et al.*, 2000; Song *et al.*, 2020). It is also a prominent reactive oxygen species (ROS) scavenger, thus preventing oxidative disruption to the electron transport chain through inhibition of hydroxyl radicals (Akashi *et al.*, 2001; Yokota *et al.*, 2002). Synthesis of citrulline through ornithine–arginine interconversion is mediated in the chloroplast in response to plant N status, and prompts crosstalk between the chloroplast, mitochondria, and the cytosol to maintain N homeostasis (Urbano-Gámez *et al.*, 2020; Chen *et al.*, 2022). These reactions would take place in the chloroplast-rich mesophyll of C_3_ plants, causing the amino acids to diffuse through the BS to reach the phloem stream for export (Leegood, 2008). In contrast, if the same principle is applied to C_2_ plants, then synthesis of N-rich amino acids will also be common in their chloroplast-rich BS cells (Khoshravesh *et al.*, 2016), which would conveniently expedite N transport (Leegood, 2008). Maintaining functional chloroplast activity in both mesophyll and BS cells should theoretically permit flexibility of ornithine or arginine synthesis between the two cell types in C_2_ species. This versatility may decline as mesophyll chloroplast concentrations wane with the emergence of a C_4_ phenotype in some lineages (e.g. Stata *et al.*, 2016). The role of arginine may be particularly fundamental to N distribution as it has a high N:C ratio, which is useful for N storage and transport under abiotic stress (Winter *et al.*, 2015; Blume *et al.*, 2019). Ornithine, arginine, and citrulline levels are thought to increase under high photorespiratory flux to reduce NH_3_ toxicity, promote photoprotection, provide drought mitigation, and improve NUE (Joshi and Fernie, 2017). This suggests that C_2_ phenotypes may strategically balance their C:N ratio through these three amino acids, uniting a TCA-derived C skeleton with photorespired NH_3_ for glutamate synthesis prior to conversion.

**Fig. 4.**
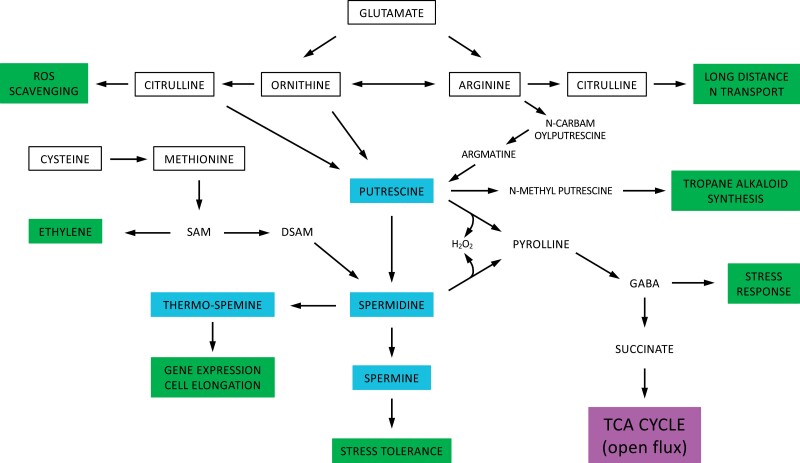
Polyamine synthesis from glutamate showing relevant outcomes, noted at each stage (green). Input from cysteine is also shown. Amino acids are outlined in black and polyamines are shown in blue (tropane alkaloid synthesis may only be relevant to C_2_ species in the *Brassicaceae*).

The decarboxylation of ornithine or arginine by the enzymes ornithine decarboxylase and arginine decarboxylase, respectively, is the first catalytic step in the process of polyamine synthesis (Fig. 4). Polyamines such as putrescine, spermidine, and spermine are important in plants to stimulate DNA synthesis, growth, and physiological development (Chen *et al.*, 2019). However, they are also known to play an important role as substantial N sinks and C:N regulators through hydrogen peroxide signalling (Moschou *et al.*, 2012). Additionally, polyamines are thought to improve abiotic stress tolerance, as their levels increase in response to high salinity, high or low temperatures, and drought (Tiburcio *et al.*, 2014; Liu *et al.*, 2015) (Fig. 4). Two forms of polyamines are found in plants, free amines and amide conjugates, such as hydroxycinnamic acid amides (Tiburcio *et al.*, 2014). Wild rocket (*D. tenuifolia*), a C_2_ crop species, has been found to up-regulate the N-rich arginine metabolism under stress conditions, which very well could serve as an effective N distribution pathway whilst supporting a stress tolerance strategy (Cavaiuolo *et al.*, 2017). Its roles in supplying export pathways and in being a precursor to stress-relevant pathways supports the idea that BS NH_3_ functions as a leaf N hub from which the release of NH_3_ into the BS has many beneficial outcomes. This view is in contrast to previous suggestions that NH_3_/NH_4_^+^ is a problem that must be solved by shuttling it back to the mesophyll (Mallmann *et al.*, 2014).

Plants have evolved various methods to detoxify both NH_3_ and NH_4_^+^, such as reformation into non-toxic derivatives or vacuole sequestration. NH_3_ detoxification in C_2_ plants may use GDH, either exclusively or co-dependently, alongside the ornithine pathway as previously described (Fig. 3B). These methods are also thought to be active under high levels of NH_4_^+^ in C_3_ plants (Bittsánszky *et al.*, 2015). Therefore, under environmental conditions that promote a high photorespiratory flux and generate NH_4_^+^, the C_2_ phenotype may have evolved a highly efficient detoxification approach. Studies on C_3_ plants have found increased expression of the GDH enzyme in both leaves and roots under high photorespiratory conditions, indicating an important role in NH_3_ sensing and whole-plant tolerance (Turano *et al.*, 1997; Cruz *et al.*, 2006; Skopelitis *et al.*, 2006; Sarasketa *et al.*, 2014). If the activation energy requirements were low enough, this enzyme could be a prominent player in NH_3_ detoxification by a cytosolic isoform interconverting 2-OG and glutamate. The process may also present in a cyclic form, interacting with GS/GOGAT, as suggested by Stitt *et al.* (2002) (Fig. 3A). This would regulate both glutamate pools and 2-OG TCA levels, maintaining C flow through the non-cyclic TCA chain, with 2-OG being the predominant C acceptor for NH_3_ (Stitt *et al.*, 2002). Glutamate would then present either as a precursor for other amino acids or for vascular export, maintaining a constant pool size. Interestingly, isoenzymes of both GS and GDH convey metabolic flexibility and spatial variability for adaptation to environmental and developmental changes under varying N nutrition to optimize growth (Fontaine *et al.*, 2009). Evidence of GDH has been found not only in the cytosol in response to high NH_3_ levels, induced by photorespiration, but also in the companion cells of the vasculature, especially in the midrib (Tercé-Laforgue *et al.*, 2004). Therefore, the increase in vein density initiated at the very early stages of C_4_ photosynthesis evolution (Christin *et al.*, 2013; Griffiths *et al.*, 2013) could primarily be for rapid NH_3_ detoxification, with any hydraulic improvement being a secondary benefit (Sack and Holbrook, 2006).

One of the side effects associated with photorespiration is the stimulation of nitrate uptake (Bloom *et al.*, 2010) (Fig. 3). As mentioned earlier, the C_2_ salad crop *D. tenuifolia* often contains dangerously high leaf nitrate levels that must be closely monitored by growers prior to harvest (Weightman *et al.*, 2012). The crop itself benefits from this accumulation because the influx of nitrate is a sink for excess energy dissipation under high light conditions, as the reduction of nitrate has a high energy requirement of eight electrons initially provided by NAD(P)H as reductant (Long *et al.*, 2015). Storage of nitrate in the vacuole also helps to maintain the osmotic status of a leaf, which would be particularly important under hot and dry conditions where photorespiratory fluxes will be high (Bloom, 2015). This hyperaccumulation of nitrate causes an alkaline pH within the cell. To address this, phosphoenolpyruvate carboxylase (PEPC) and malate dehydrogenase (MDH) are stimulated to produce higher levels of malate to neutralize the pH (Stitt *et al.*, 2002). This malate would then be transported into the roots for decarboxylation in C_3_ species. However, in C_4_ species, the malate would be decarboxylated in the BS to enhance the C concentration there. From a C_2_ biochemistry perspective, this suggests that the original purpose of malate decarboxylation in a proto-C_4_ BS may be to address cellular pH homeostasis following excessive nitrate uptake under photorespiration. Indeed, when photorespiration is absent, a plant would have to devote a quarter of photosynthate to nitrate uptake and reduction (Bloom, 2015).

## Sulfur assimilation

**Fig. 5.**
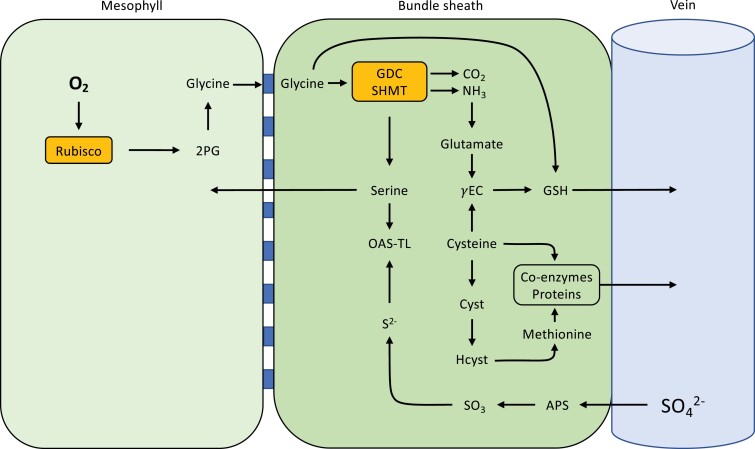
Proposed sulfate assimilation in C_2_ species. Sulfate is taken up from the soil and delivered to the bundle sheath via the xylem. Glycine and γ-glutamylcysteine (γEC) form glutathione (GSH), a vital plant compound for abiotic stress resistance. Cysteine and methionine are exported for other important plant compounds. Cys, cystathionine; Hcyst, homocysteine; OAS-TL, *O*-acetylserine; APS, adenosine 5ʹ-phosphosulfate.

Sulfur (S) is an essential nutrient for plant growth that is integrally entwined within the C and N metabolic framework. S assimilation is directly linked to photosynthetic capacity and consequently photorespiration, owing to the effect of S metabolism on redox regulation and electron consumption (Abadie and Tcherkez, 2019). Photorespiration plays a major role in supplying serine to the plant by stimulating S uptake (Abadie and Tcherkez, 2019). In turn, this influx of serine under conditions that promote photorespiration increases supply of C_1_ units to provide the basic components needed for amino acids such as methionine and cysteine (Fig 5), which are essential for crops with high demand for these metabolites, such as those in the *Brassicaceae* for glucosinolate synthesis (Koprivova and Kopriva, 2016). In fact, there are three major routes for serine synthesis: through photorespiration, glycerate, and the phosphorylated pathway (Anoman *et al.*, 2019), with photorespiration being the dominant serine source in both C_3_ and C_2_ leaves. Serine plays a fundamental role in cellular processes, as the cytosolic serine pool is the principal source of C_1_ units for synthesis of methionine and *S*-adenosylmethionine (AdoMet). AdoMet is the predominant methyl donor for many methylation reactions such as lignin biosynthesis, DNA methylation, and other crucial processes (Fernie *et al.*, 2013). Therefore, through serine, photorespiration supports at least four essential plant processes.

Serine biosynthesis will be different in C_4_ compared with C_2_ or C_3_ plants. The reduced photorespiratory flux in C_4_ leaves will require serine biosynthesis to be re-routed, either through its import via a different organ, the glycerate pathway, or via the phosphorylated pathway of serine synthesis (Ros *et al.*, 2014; Zimmermann *et al.*, 2021). Notably, serine synthesis has been found to be transferred to the root when photorespiration is reduced in C_4_*Flaveria* (Gerlich *et al.*, 2018). Alternatively, the glycerate pathway may provide serine for C_4_ plants, which could be afforded by their increased C assimilation and crucial for their environmental stress tolerance (Igamberdiev and Kleczkowski, 2018).

The role of serine in the C_2_ BS could be a particularly vital one, as serine acts as the precursor for cysteine synthesis, coupled with a reduced sulfide ion (Takahashi *et al.*, 2011). The location of C_2_ serine synthesis within the BS could improve the efficiency of the signalling mechanism required for cysteine application in the coordination of stress responses, as its close proximity to the vein would allow for rapid export. Cysteine acts as the S currency for all plant primary and secondary compounds, including DNA, amino acids, proteins, and defence compounds (Abadie and Tcherkez, 2019), and functions as the precursor for the photoprotective glutathione and for methionine for protein synthesis (Kopriva *et al.*, 2019) (Fig. 5).

Glutathione is known to accumulate under intensive light levels to facilitate acclimation and mitigate photo-damage (Speiser *et al.*, 2015). The primary role of glutathione is as a principal ROS scavenger, and it also plays a significant role in redox signalling and enzyme regulation (Kopriva *et al.*, 2019; Li *et al.*, 2020). To produce glutathione, three amino acids—cysteine, glutamate, and glycine—are amalgamated in a two-part reaction, catalysed by γ-glutamylcysteine synthetase and glutathione synthetase (Zechmann, 2014) (Fig. 5). Glutathione then pairs with ascorbate in an interactive pathway to mitigate ROS damage to organelles and photosynthetic machinery under stress conditions (Foyer and Noctor, 2011; Zechmann, 2014). Consequently, each cell is then dependent on glutathione for initiating defence gene activation through redox signalling. Synthesis of glutathione is under regulation of the hormone abscisic acid and is often induced by drought stress. Crucially, glutathione homeostasis is needed for compartment-specific plant defence sensing and signalling, especially within the chloroplast (Hasanuzzaman *et al.*, 2017). Appropriate application of this process in C_2_ species is found when the C_2_ mechanism is activated under photorespiratory conditions, as the BS glycine pool can provide one of the substrates for glutathione by siphoning a fraction prior to serine conversion (Fig. 5). Interestingly, glutathione concentrations show a gradual increase along the C_3_ to C_4_ evolutionary continuum in the genus *Flaveria* (Kopriva, 2011).

## Carbon, nitrogen, and sulfur integration

**Fig. 6.**
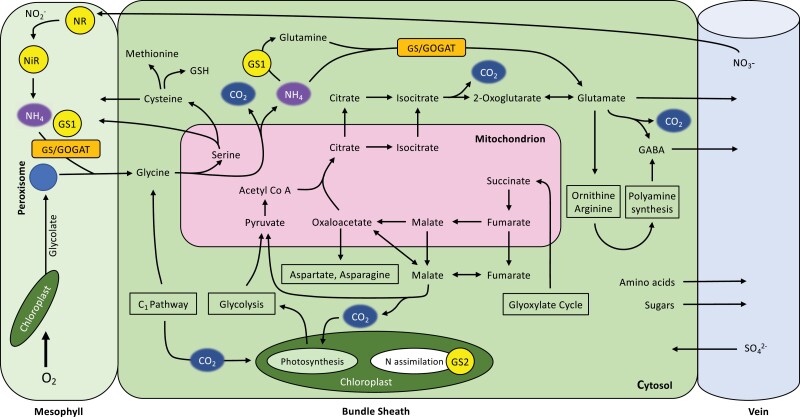
Proposed integration of carbon, nitrogen, and sulfur metabolisms in C_2_ species. A hypothetical C_2_ mechanism showing integration of carbon, nitrogen, and sulfur metabolic pathways in the enlarged bundle sheath, including alternative pathways for photorespired NH_4_. Yellow circles denote glutamine synthetase isoforms: GS2 found in the chloroplast and the cytosolic GS1.

Photorespiration, and by extension the C_2_ glycine shuttle, acts as an important metabolic junction with many of its intermediates linking to other pathways that are central to primary metabolism (Fig. 6). In this section, we discuss how the metabolisms of C, N, and S discussed above are integrated into a central hub through photorespiration.

In C_3_ species, C metabolism is tightly linked to N pathways, with nitrate and NH_3_ assimilation at the forefront. Changes in nitrate uptake and influxes of NH_3_ affect the transcription of metabolic enzymes, such as PEPC, pyruvate kinase (PK), citrate synthase (CS), and isocitrate dehydrogenase (ICDH) (Scheible *et al.*, 2000). Adjustments to these enzymes consequently allow for the cellular pH to be balanced in relation to nitrate uptake and C diversion, owing to the poor buffering capacity of the leaf (Stitt *et al.*, 2002). Interactions between C and N can therefore be mediated appropriately by the TCA pathway in response to metabolite signalling to prioritize the cellular C:N balance. The uptake of nitrate can also have profound effects on cellular metabolism via the down-regulation of the AGPS gene, which encodes the starch synthesis regulatory subunit enzyme, ADP-glucose pyrophosphorylase (Scheible *et al.*, 2000) and leads to an increase in PEPC activity and organic acids. Perhaps the localization of nitrate reduction to the mesophyll leads to an increase in PEPC activity that confines starch synthesis to the BS of C_4_ species. This observation of C partitioning in C_4_ species has been recently reviewed by Furbank and Kelly (2021). In C_2_ species, however, the partitioning of photorespiration between the mesophyll and BS could potentially invoke consequences for the energy consumption needed for nitrate reduction, with NAD(P)H supplied by either the chloroplast or mitochondria depending on environmental conditions (Gardeström and Igamberdiev, 2016). One option would be that in high light environments, NAD(P)H would be amply supplied by mesophyll chloroplasts, despite nitrate reductase activity taking place. This relies on chloroplasts being interactive with other organelles, with cellular stoichiometry being flexible especially under stress or fluctuating conditions. Alternatively, TCA pathway activation in the light would also be another source of ATP under high stress conditions, alongside other ATP-generating processes within the mesophyll cellular metabolism, contributing to energy balancing (Noctor and Foyer, 1998).

Efficient functioning of diurnal respiration could equip C_2_ species to optimize photorespired NH_3_ by integrating glutamate with C skeletons of anaplerotic organic acids for amino acid formation, with an integral flexibility of a dual branched TCA pathway to adjust to cellular C:N status in response to NH_3_ influx and other forms of stress (Fig. 6) (Vega-Mas *et al.*, 2019; and see Ji *et al.*, 2020 for a similar mechanism in response to heavy metal stress). Interestingly, verification of a diurnal TCA pathway was found in the C_2_ species *Salsola divaricata* by Gandin *et al.* (2014) who successfully showed the C_2_ mechanism and day respiration working in tandem to acclimate the plant to both high and low temperatures. There is a necessity for the TCA cycle to operate a diurnal open branched system to mitigate degradation of α-ketoglutarate dehydrogenase and succinyl-CoA under oxidative stress conditions (Sweetlove *et al.*, 2002). This is supported by evidence that mitochondria can adapt their metabolism in accordance to variation in light intensity to coordinate responses with those seen in photorespiration (Huang *et al.*, 2013; Reinholdt *et al.*, 2019).

A C_2_ solution could be an open flux TCA flow straddling the mitochondrial–cytosolic membrane, using cytosolic isoenzymes to offer flexibility for metabolite pools to compensate for redox regulatory reactions and address the cellular C:N balance (Fig. 6). Although this strategy is sometimes employed by certain C_3_ plants, operating at a low to moderate flux, to improve C-use efficiency in specific environments (Tcherkez *et al.*, 2017), C_2_ phenotypes may depend on optimization of the diurnal respiratory pathway to increase C and balance the C:N ratio for acclimation as photorespiration occurs under challenging conditions. This may even be a characteristic of C_2_ or C_4_ species with the potential to adjust diurnal respiration at a higher flux in dynamic response to environmental conditions in comparison with C_3_ species. This is supported by the idea of a diurnal TCA pathway being regulated by a CO_2_/O_2_ ratio (i.e. rates of photosynthesis and photorespiration in response to C and NH_3_ flux), and a direct correlation between high photorespiration flux and an increase in day respiration (Tcherkez *et al.*, 2008). If C_2_ species partially relocate the TCA reactions to the cytosol, as some C_3_ species do, then this could potentially increase the efficiency of signalling cascades for stress response mechanisms and source–sink transport given its close proximity to the vein in C_2_ plants. This strategy may also improve metabolic coordination between photosynthesis, respiration, photorespiration, and N assimilation in response to hostile environments, a synchronicity that could be a requirement to enhance biochemical adjustments along the C_3_ to C_4_ evolutionary trajectory, with N acquisition at the forefront.

Within the mesophyll cells of C_3_ species, the arrival of N that has been assimilated through glutamate is regulated by γ-aminobutyric acid (GABA), with subsequent Ca^2+^ and GABA signalling (and metabolism) to minimize disruption to the synthesis of amino acids, DNA, chlorophyll, and secondary metabolites under abiotic stress and utilize photorespired C in amino acids (Nunes-Nesi *et al.*, 2010; Busch *et al.*, 2018). GABA is synthesized in the cytosol from glutamate by glutamate decarboxylase. On its return to the mitochondria, GABA is transaminated by GABA transaminase to succinic semialdehyde. It is finally oxidized by succinate semialdehyde dehydrogenase to form succinate to rejoin the TCA pathway. This process incorporates glutamate into GABA, storing N before joining the TCA pathway, to recycle the C framework and help balance the C:N ratio (Bouche and Fromm, 2004) (Fig. 3B) (GABA can also be derived from polyamines, as shown in Fig. 4). Following the incorporation of succinate into the TCA pathway, it is then converted by succinate dehydrogenase to fumarate, and from fumarate to malate by fumarase. Indeed, a 3-fold elevation of fumarase was found to be present in the BS of the C_2_ species *Moricandia arvensis* (Rawsthorne, 1988), which could indicate the presence of a GABA mechanism (i.e. the GABA shunt) under photorespiratory conditions that functions to continue TCA pathway operation and boost malate pools in response to stress and NADH ratios (Igamberdiev, 2020). The inclusion of GABA synthesis to boost fumarase activity could be an important mechanism, as a reduction of this enzyme leads to photosynthesis inhibition by defective stomatal movement (Nunes-Nesi *et al.*, 2007).

In C_3_ species, elevated GABA levels stimulate the cell’s stress response for photoprotection, osmoregulation, C:N balancing, regulation of stomatal opening, and gibberellic acid synthesis (Li *et al.*, 2021). The molecule is known to be a thermo-protectant that can improve C assimilation under heat stress and maintain leaf water levels (Priya *et al.*, 2019). It also has a role in root proliferation and primary root growth (Li *et al.*, 2021), which would aid in water and nutrient acquisition. GABA can be synthesized by the citrate branch of the dual branched TCA pathway in the light (Fig. 6). The TCA pathway can provide either energy or backbones for amino acid synthesis, in response to C:N balance status, by switching flux modes between the dual branches of either the citrate or malate lines (Figs 2B, 6), with the conventional cyclic mode more likely to be used in darkness (Vega-Mas *et al.*, 2019). Within C_3_ plants, operation of the malate and citrate valves of the diurnal TCA pathway forms a dynamic coordination between chloroplast and mitochondria, which maintains energy balance through redox regulation, with chloroplastic malate acting as a redox signal of imbalance within the cell (Scheibe, 2019). Meanwhile, the mitochondrial citrate valve can also support organic acid synthesis and N assimilation in the cytosol in accordance with cellular demand, resulting in regulation of gene transcription (Igamberdiev, 2020). C_2_ species may utilize this approach, where straddling both mitochondria and cytosol would allow for greater metabolic speed and efficiency within the BS (Fig. 6). Additionally, the synthesis of ATP produced in the mitochondria will also have a direct influence over subsequent transcription and translation of organelle genomes as a response to energy status needed for metabolic and assimilatory co-dependence (Liang *et al.*, 2015).

While the coordination of regulatory mechanisms for C, N, and S assimilation remains undefined in the literature, its importance is clear, as each element is crucial for cysteine and glutathione synthesis to provide ROS protection through redox signalling and regulation in tandem with an NH_3_–glutamate feed to prevent NH_3_ accumulation. Indeed, both N and S uptake and assimilation are improved when NH_3_ is the N source, implying a link between these metabolic pathways (Coleto *et al.*, 2017), especially under hostile environments, thus implicating the involvement of photorespiration (Eisenhut *et al.*, 2015).

C_2_ biochemistry may provide enhanced protection to the plant. The high flux through photorespiration that C_2_ plants experience will also create high fluxes in many metabolites required for secondary defence compounds, such as flavonols and glucosinolates, when mitigating for excessive heat, drought, and oxidative stress (Nakabayashi *et al.*, 2014; Essoh *et al.*, 2020). Indeed, these secondary defence compounds have been found in two C_2_*Brassicaceae* species that lack C_4_ relatives—*D. tenuifolia* and *Moricandia arvensis* (Martínez-Sánchez *et al.*, 2007; Marrelli *et al.*, 2018). Moreover, the rich Ca^2+^ content and high levels of antioxidants, polyphenols, glucosinolates, alkaloids, and amino acids found in *D. tenuifolia* leaves suggest that GABA synthesis could be in operation to protect the plant (Brock *et al.*, 2006; Bell *et al.*, 2015). Secondary metabolites rich in N and S are not confined to the *Brassicaceae.* High levels of polyphenols, glycosides, alkaloids, etc. have also been identified in the C_2_ species *Parthenium hysterophorus* (*Asteraceae*) and *Mollugo verticillata* (*Molluginaceae*), both of which, interestingly, are highly invasive and also lack C_4_ relatives (Lundgren, 2020; Kumar and Shariff, 2021; Jaiswal *et al.*, 2022). Lineages that do synthesize these compounds would need to increase both C gain and TCA flux to provide a C framework over and above that needed for growth and reproduction. Within C_2_ plants, *de novo* nitrate uptake must also increase to supplement photorespired NH_3_ fixation in order to maintain plant growth while also synthesizing the N-containing secondary compounds. Of course, these interesting metabolites are also present in many C_3_ species, providing nutrient-rich crops and plant stress resistance.

If C_2_ species use their consistent photorespiratory flux to balance incoming C, N, and S with their secondary metabolite sink, on top of that which is already required for primary metabolism within their environment, then that would be sufficient to trap these lineages in a C_2_ state. Progression towards C_4_ would limit photorespiration and therefore decrease the availability of substrates for defence and mitigation pathways. A stable C:N balance within a certain criterion of environmental conditions could allow C_2_ phenotypes to thrive by facilitating enhanced plasticity between its C_3_ and C_2_ cycles. Under conditions that promote a high photorespiratory flux, the C_2_ mechanism could feed into an N sink for protective secondary metabolites, which could then be stored within the vacuole. Under non-photorespiratory conditions, the C_3_ cycle would be sufficient to provide an adequate C:N ratio to fuel growth and reproduction within a C_2_ plant. This would of course depend on the ability for C_2_ cycle engagement to self-optimize under variable environmental conditions that could oscillate dramatically.

Photorespiration serves to dissipate energy (Eisenhut *et al.*, 2019) and therefore provides protection against ROS. The much lower photorespiratory flux experienced by C_4_ plants would consequently require an alternative metabolism to enable crosstalk between organelles to reach optimal protection and synthesize secondary compounds for plant protection.

## Implications for stress tolerance in C_2_ plants

Our proposed integration of C, N, and S metabolism in C_2_ plants (Fig. 6) is underpinned by the diurnal, dual branched TCA pathway being able not only to function, but also to optimize and coordinate responses between photosynthesis, respiration, photorespiration, and ROS mitigation. We suggest that confinement within the BS cytosol and mitochondria is key to the success of C_2_ species. Through this novel organization of primary metabolism, a C_2_ crop should theoretically be able to maintain yields and nutritional integrity better than a C_3_ crop as CO_2_ levels rise under the advancement of climate change. An efficient, integrated optimization of metabolic pathways, supplemented by retaining C_3_ gene expression in mesophyll cells, would let the CBB cycle, TCA pathway, N metabolism, and S metabolism operations be maintained under temperate conditions, giving C_2_ plants the flexibility to withstand cooler conditions and low light levels better than C_4_ plants (Bellasio and Farquhar, 2019). Indeed, C_2_ plants occur in cooler regions despite an overall preference for warmer climates (Lundgren and Christin, 2017), displaying evidence of C_2_ climatic versatility. C_2_ plants may have alternative routes of photorespiratory metabolism, such as another open flux system that could replenish glycine and serine pools for auxiliary metabolic processes (Timm *et al.*, 2012; Busch, 2020). There may also be multiple photorespiratory phenotypes within a species, as has been identified in the C_3_ model species *Arabidopsis thaliana* (Timm *et al.*, 2012). If also present in C_2_ species, this intraspecific photorespiratory diversity would facilitate tolerance of unfavourable or fluctuating environments. Indeed, under specific combinations of light and temperature, C_2_ species are predicted to simultaneously optimize CO_2_ assimilation and resource use (Blätke and Bräutigam, 2019; Johnson *et al.*, 2021). This efficiency therefore reduces evolutionary pressure and facilitates the stable state version of the C_2_ phenotype.

## Conclusion

For a C_2_ lineage to transition towards C_4_, evolution needs to find a solution for serine supply, for the sufficient production of export amino acids in the absence of high flux through NH_3_, and potentially for substrate production to maintain—even at a lower level—synthesis of metabolites necessary for abiotic stress resistance. The primary advantage of the C_2_ phenotype may therefore be efficient NH_3_ detoxification in response to increased photorespiratory flux. With a rapid influx of N, the plant would require improvements in C backbone synthesis to provide a C framework for protective secondary metabolites in addition to amino acids. Therefore, the strong need for an effective N sink would take priority over C assimilation under photorespiratory conditions, for rapid N remobilization and stress protection under conditions that incite high photorespiratory flux. Indeed, plant lineages that operate pathways to successfully integrate N storage and transport (e.g. via increased investment in the shikimate or ornithine pathways) may be more likely to adopt the C_2_ phenotype on the strength of their N sink. Based on the competence of the N sink, our theory may explain why some C_2_ lineages do not transition to a C_4_ state (i.e. the stronger the N sink, the more stable the C_2_ state becomes). These C_2_ populations could thrive under nutritionally stable soil types and may have co-evolved alongside C_4_ species to optimize the C_2_ pathway towards a ‘super C_2_’ phenotype. For species that do not specialize in synthesizing N-rich compounds, their alternative may be to increase amino acid production and consequently require more C, resulting in the amino acid shuttling we associate with C_4_ phenotypes. Interestingly, this theory gives secondary metabolites a more prominent role within plant biochemistry and evolution, in addition to their well-known role in stress response. Importantly, a high performing version of C_2_ metabolism may provide a lifeline for our crop resources under climate change as a solution to compromised food security and quality.

## Abbreviations

BS: bundle sheath
CBB: Calvin–Benson–Bassham cycle
CCM: carbon-concentrating mechanism
GDC: glycine decarboxylase complex
GDH: glutamate dehydrogenase
GS/GOGAT: glutamine synthetase and glutamine:2-oxoglutarate aminotransferase
NUE: nitrogen use efficiency
PEPC: phosphoenolpyruvate carboxylase
PK: pyruvate kinase
ROS: reactive oxygen species
RuBP: ribulose 1,5-bisphosphate
SHMT: serine hydroxymethyl transferase
TCA: tricarboxylic acid
